# Skipping breakfast, overconsumption of soft drinks and screen media: longitudinal analysis of the combined influence on weight development in primary schoolchildren

**DOI:** 10.1186/s12889-018-5262-7

**Published:** 2018-03-16

**Authors:** Meike Traub, Romy Lauer, Tibor Kesztyüs, Olivia Wartha, Jürgen Michael Steinacker, Dorothea Kesztyüs, the Research Group “Join the Healthy Boat”, Ileana Briegel, Jens Dreyhaupt, Eva-Maria Friedemann, Anne Kelso, Lina Hermeling, Eleana Georgiou, Ekaterine Goosmann, Christine Lämmle, Rainer Muche, Olga Pollatos, Luise Steeb, Belinda Hoffmann, Susanne Kobel, Tamara Wirt

**Affiliations:** 1 Medical Center, Division of Sports and Rehabilitation Medicine, University of Ulm, Frauensteige 6, Haus 58/33, 89075 Ulm, Germany; 2 Institute of General Medicine, Ulm University, 89081 Ulm, Germany; 3 Department of Computer Science, Ulm University of Applied Sciences, 89081 Ulm, Germany; 4 Institute of Medical Systems Biology, Ulm University, 89081 Ulm, Germany

**Keywords:** Child, Soft drink, Breakfast, Screen media, Overweight, Obesity, Prevention & control

## Abstract

**Background:**

Regular breakfast and well-balanced soft drink, and screen media consumption are associated with a lower risk of overweight and obesity in schoolchildren. The aim of this research is the combined examination of these three parameters as influencing factors for longitudinal weight development in schoolchildren in order to adapt targeted preventive measures.

**Methods:**

In the course of the Baden-Württemberg Study, Germany, data from direct measurements (baseline (2010) and follow-up (2011)) at schools was available for 1733 primary schoolchildren aged 7.08 ± 0.6 years (50.8% boys). Anthropometric measurements of the children were taken according to ISAK-standards (International Standard for Anthropometric Assessment) by trained staff. Health and lifestyle characteristics of the children and their parents were assessed in questionnaires. A linear mixed effects regression analysis was conducted to examine influences on changes in waist-to-height-ratio (WHtR), weight, and body mass index (BMI) measures. A generalised linear mixed effects regression analysis was performed to identify the relationship between breakfast, soft drink and screen media consumption with the prevalence of overweight, obesity and abdominal obesity at follow-up.

**Results:**

According to the regression analyses, skipping breakfast led to increased changes in WHtR, weight and BMI measures. Skipping breakfast and the overconsumption of screen media at baseline led to higher odds of abdominal obesity and overweight at follow-up. No significant association *between soft drink consumption and weight development was found.*

**Conclusion:**

Targeted prevention for healthy weight status and development in primary schoolchildren should aim towards promoting balanced breakfast habits and a reduction in screen media consumption. Future research on soft drink consumption is needed. Health promoting interventions should synergistically involve children, parents, and schools.

**Trial registration:**

The Baden-Württemberg Study is registered at the German Clinical Trials Register (DRKS) under the DRKS-ID: DRKS00000494.

## Background

The increase in overweight and obesity in children and adolescents as a worldwide health problem [1] has led the World Health Organization (WHO), in the introduction of its World Health Report, to define overweight and obesity as one of the future challenges [2]. In particular, childhood obesity has the longer-term risk that overweight or obese children become overweight adults and develop, e.g., cardiovascular diseases, diabetes or orthopaedic problems [3]. Overweight and obesity are most often the result of an unhealthy lifestyle, leading to a rising prevalence of non-communicable diseases (NCD) [4]. It is assumed that abdominal obesity has the most risky influence on the development of NCDs [5]. There are multiple and complex reasons for overweight and obesity. However, in addition to genetic and physiologic aspects, lifestyle patterns are the most frequent causes of weight gain. In particular, a sedentary lifestyle with a lot of screen media consumption and reduced physical activity [6, 7] skipping breakfast [8], and a high energy intake, e.g. overconsumption of high-calorie soft drinks [7] seem to be relevant factors for weight gain and the development of overweight in primary schoolchildren. Systematic reviews show that in schoolchildren skipping breakfast is associated with an increase in body mass index (BMI) and a higher risk of becoming overweight or obese [8, 9]. In addition, there exists the general view that prolonged use of screen media is associated with childhood obesity [10]. On the one hand, time spent with screen media leads to physical inactivity, and on the other hand, it contributes to an increased energy intake through snacking and consuming soft drinks in front of the screen [10]. A study of Krahnstoever Davison et al. shows that 7-year-old girls who exceed the recommendations of the tolerable time watching TV are more likely to be overweight at age 11 [11]. Due to the high calorific density of soft drinks, there is a special interest in the association of soft drink consumption and obesity [12]. Two recent reviews conclude that the consumption of soft drinks is related to obesity [13, 14]. Additionally, the association between soft drink consumption and various weight parameters is consistent [15]. For example, Lee et al. confirm a link between high soft drink consumption and higher waist circumference (WC) and BMI z-scores [16].

The aim of the present study is to investigate the longitudinal associations of skipping breakfast, the consumption of soft drinks, and screen media as combined factors for longitudinal weight development in schoolchildren. New information for multicomponent and targeted interventions for obesity prevention in schoolchildren could be derived from these findings.

## Methods

### Study design

The Baden-Württemberg Study is a prospective, cluster-randomized and longitudinal study with a waitlist control group to evaluate the school-based health promotion programme “Join the Healthy Boat”. The programme is included in the curriculum of grades one to four at primary schools in Baden-Württemberg, south-west Germany. A detailed description of the evaluation design and the programme can be found elsewhere [17]. The aim of the programme is to support children to develop a healthy lifestyle in the terms of physical activity, reduction in consumption of soft drinks and in screen media. Behavioural and environmental components are combined equally. In order to analyse the success of the programme and its effects, data collection was conducted for baseline measurements in autumn 2010, and for follow-up in autumn 2011.

### Ethics, consent and permissions

Besides the agreement of schools and teachers to participate in the study, parents had to give their written, informed consent for their child. The trial protocol was approved by the ethics committee of Ulm University (Application No. 126/10). The Baden-Württemberg Study is registered at the German Clinical Trials Register (DRKS) under the DRKS-ID: DRKS00000494.

### Participants and data

At baseline and follow-up, data from 1733 children from first and second grade was collected. Anthropometric data of the children such as height, weight, and waist circumference were assessed in schools by trained staff. Data from parental questionnaires was available for 1545 children (89%) at baseline and follow-up. Parents gave information about their own anthropometric data as well as health and living conditions. They also provided details about their child’s health behaviour, lifestyle and socioeconomic background.

### Demographics

The parental education level was assigned on the basis of the CASMIN classification (Comparative Analysis of Social Mobility in Industrial Nations) [18], and family education level was defined as the highest level of two parents or a single parent. Family education level was dichotomized for analysis into elementary and intermediate level, on the one side, compared with tertiary level on the other side. A child’s migration background was defined as at least having one parent being born abroad, or at least one parent having mainly spoken a foreign language and not German during the child’s first years of life. Household income was assessed according to the categories used in the KiGGS survey (German Health Interview and Examination Survey for Children and Adolescents) [19] and dichotomized for analysis into two groups: Families with a household income of €1750 or less per month, and families with more than €1750 per month.

### Health and lifestyle characteristics

Parents were asked to give information on their children’s health and health behaviour. Relevant questions were taken from the validated questionnaires of the German KiGGS survey [20]. Frequency of consuming soft drinks at school and outside school (several times a day, every day, several times a week, once a week, less than once a week, never) was assessed on a 6-point Likert scale. Time spent with screen media on school days and at weekends, as well as playing outside (never, about 30 min/day, about 30–60 min/day, about 1–2 h/day, about 2–3 h/day, about 3–4 h/day, more than 4 h/day) was assessed on a 7-point Likert scale. Variables were dichotomized for analysis (soft drinks > 1/week, playing outside > 60 min/day, screen media > 1 h/day). On a 4-point Likert scale, parents stated how often their children ate breakfast before going to school. The answers also were dichotomized: “Never and rarely” versus “often and always”. Furthermore, they stated the number of days per week during which their children were physically active at a moderate to vigorous level for at least 60 min a day, as recommended by the World Health Organization (WHO) [21]. This item was dichotomized for analysis at the middle category (physically active ≥4 days/week ≥60 min/day). Moreover, parents were asked whether and how long their children were breastfed, and whether their mother had smoked during pregnancy. Finally, parents stated self-assessed information about their height, weight and WC, from which their weight status could be derived.

### Anthropometric measurements

Trained staff took the anthropometric measurements of the children according to ISAK-standards [22]. Height was measured to the nearest 0.1 cm and body weight was assessed to the nearest 0.1 kg using a stadiometer (Stadiometer, Seca®, Germany) and an electric calibrated and balanced scale (Seca®, Germany). WC was measured midway between iliac crest and lower costal arch to the nearest 0.1 cm using a flexible metal tape (Lufkin Industries Inc., Texas, USA). The children’s BMI was computed as weight divided by height squared (kg/m^2^). According to German reference data, cut-off points for overweight children were set above the 90th age- and gender-specific BMI percentile; for obese children above the 97th percentile [23]. WC divided by height in centimetres was used to calculate the waist-to-height-ratio (WHtR). According to Ashwell & Hsieh, participants with a WHtR ≥0.5 were categorized as abdominally obese [24]. Parental BMI was determined based on the self-reported weight and height data from the questionnaires and was categorized as overweight (BMI ≥ 25.0) or obese (BMI ≥ 30.0) [25]. Parental WHtR was calculated as the ratio of self-reported WC to height in centimetres, and the cut-off point for abdominal obesity was defined as WHtR ≥0.5 [24].

### Missing data

In observational studies the problem of missing data often occurs, possibly leading to biased results [26]. Therefore, baseline differences between cases with and without missing values for the final regression model were statistically tested and reported.

### Statistical analysis

Group differences in baseline data between boys and girls, as well as between participants with and without missing values, were tested. The Mann-Whitney-U test was used for continuous data, and Fisher’s exact test for categorical data. Statistical analyses were performed using the statistical software packages IBM SPSS Release 21.0 for Windows (SPSSInc, Chicago, IL, USA) with a significance level set at α = 0.05 for two-sided tests.

To account for the clustering of data in schools, generalised linear mixed effects models were calculated for the prevalence of abdominal obesity, overweight and obesity at follow-up. Changes in WHtR, weight in kg and BMI measureswere analysed in linear mixed effects regression analyses. Variables from models derived in previous investigations were included in the analyses [27, 28]. The variables of interest were included in the respective model for each outcome parameter and were tested for their significance. Because of multiple testing and the accumulation of α-error, a Bonferroni-Holm correction was applied [29]. For this purpose, the ascending ordered quantity k (= number of single hypotheses) of the *p*-values were subjected to the rule of significance *p* < α / k, where k has been reduced by 1 in each further step.

## Results

### Baseline characteristics

Table 1 shows a summary of baseline participants’ anthropometric, health and lifestyle characteristics. Primary schoolchildren who took part in the research had a mean age of 7.08 ± 0.6 years, 50.8% of them were boys. Boys were significantly heavier and less abdominally obese than girls, but on average had a higher WC. Significantly more mothers of girls refrained from smoking than did mothers of boys Boys played outside significantly more often, reached significantly higher levels of physical activity, and spent significantly more time with screen media than did girls.Girls skipped breakfast significantly more often than did boys.

**Table 1:** Baseline characteristics of participants in the Baden-Württemberg Study (2010-2011)

	Missing	Girls	Boys	Total
	Values	(*n*=852)	(*n*=881)	(*n*=1733)
Child characteristics				
Age, years [m (sd)]		7.07 (0.64)	7.09 (0.63)	7.08 (0.63)
Migration background, n (%)	244	235 (31.6)	227 (30.5)	462 (31.0)
Control group, n (%)		371 (43.5)	407 (46.2)	778 (44.9)
Weight in kg [m (sd)]		24.45 (4.50)**	24.88 (4.82)	24.67 (4.91)
BMI, [m (sd)]		15.99 (2.19)	15.97 (2.08)	15.98 (2.14)
BMIPERC, [m (sd)]		48.96 (27.74)	48.15 (27.57)	48.55 (27.65)
Overweight, n (%)		82 (9.6)	83 (9.4)	165 (9.5)
Obesity, n (%)		30 (3.5)	38 (4.3)	68 (3.9)
WC, cm [m (sd)]		55.15 (5.91)*	55.79 (5.54)	55.48 (5.73)
WHtR, [m (sd)]		0.45 (0.04)	0.45 (0.04)	0.45 (0.04)
Abdominal obesity, n (%)		78 (9.2)*	57 (6.5)	135 (7.8)
Parental characteristics				
Single parent, n (%)	218	85 (11.3)	71 (9.3)	156 (10.3)
Maternal smoking during pregnancy, n (%)	196	65 (8.5)*	91 (11.8)	156 (10.1)
Breastfeeding, n (%)	194	651 (85.1)	535 (82.0)	1286 (83.6)
Breastfeeding months [m (sd)]	462	5.55 (3.46)	5.68 (4.05)	5.61 (3.76)
Tertiary family educational level, n (%)	269	237 (32.6)	238 (32.3)	475 (32.4)
Household income ≤ 1750 €, n (%)	381	88 (13.1)	83 (12.2)	171 (12.6)
Overweight (mother), n (%)	300	223 (31.4)	217 (30.1)	440 (30.7)
Overweight (father), n (%)	392	417 (62.8)	400 (59.1)	817 (60.9)
Abdominal obesity (mother), n (%)	788	228 (48.1)	219 (46.5)	447 (47.3)
Abdominal obesity (father), n (%)	871	325 (76.3)	317 (72.7)	642 (74.5)
Health and lifestyle characteristics				
Playing outside > 60 min/day, n (%)	248	462 (62.9)***	558 (74.4)	1020 (68.7)
Physically active ≥ 4 days/week ≥ 60 min/day, n (%)	263	161 (22.1)***	238 (32.1)	399 (27.1)
Screen media > 1 h/day, n (%)	205	86 (11.3)*	119 (15.5)	205 (13.4)
PC on school days > 1 h/day, n (%)	246	2 (0.3)**	14 (1.9)	16 (1.1)
PC at weekends > 1 h/day, n (%)	236	28 (3.7)***	72 (9.6)	100 (6.7)
TV on school days > 1 h/day, n (%)	217	89 (11.8)*	124 (16.3)	213 (14.1)
TV at weekends > 1 h/day, n (%)	228	362 (48.1)	390 (51.8)	752 (50.0)
Soft drinks > 1 time per week n (%)	197	178 (23.3)	198 (25.7)	376 (24.5)
At school, n (%)	226	57 (7.5)	52 (6.9)	109 (7.2)
Outside school, n (%)	224	174 (23.3)	192 (25.2)	366 (24.3)
Skipping breakfast, n (%)	195	116 (15.2)**	82 (10.6)	198 (12.9)

m (sd) mean (standard deviation), BMI body mass index, BMIPERC BMI percentiles, WHtR waist-to-height-ratio, WC waist circumference

*** *p*< 0.001, ** *p*< 0.01, * *p*< 0.05

### Regression analysis of changes in WHtR, weight and BMI measures

Previous investigations of the same study were taken as the basis for the regression model used here [27, 28]. A linear mixed effects regression model was formed for each outcome parameter and the variables of interest were tested for their statistical significance. Table 2 shows the longitudinal correlations of skipping breakfast, and the overconsumption of soft drinks and screen media with changes in WHtR, weight in kg and BMI percentiles, adjusted for the respective baseline measures, for socio-economic (migration background, household income, and family education level) and individual (age and gender) variables, and assignment to the intervention or control group of the underlying programme evaluation and school. Children who skipped breakfast were significantly more likely to show increases in WHtR, in weight, and in BMI percentiles.

**Table 2:** Linear mixed regression models of longitudinal changes in WHtR, weight in kg and BMI percentiles

	Changes in WHtR*^1¶^		Changes in weight*^2^ [kg]		Changes in BMI percentiles*^3^	
	(*n* = 1252)		(*n* = 1251)		(*n* = 1250)	
	*B* (SE)	*p*-value	*B* (SE)	*p*-value	*B* (SE)	*p*-value
Skipping breakfast	*0.50 (0.19)*	0.007**	*0.39 (0.12)*	<0.001***	*2.01 (0.90)*	0.027*
Soft drinks > 1 time per week	-0.01 (0.15)	0.966	-0.08 (0.09)	0.385	-0.75 (0.70)	0.282
Screen media > 1 h/day	0.29 (0.16)	0.074	0.19 (0.10)	0.054	0.70 (0.78)	0.373

*B* (SE) *B* regression coefficient (standard error), ^¶^ multiplied by 10^2^ for better interpretability, *adjusted for school, migration background, family education level, household income, age, gender, participation in the intervention, and ^1^baseline WHtR, ^2^baseline weight, ^3^baseline BMI percentiles

*** *p*< 0.001, ** *p*< 0.01, * *p*< 0.05

Skipping breakfast also influenced changes in BMI (0.21 ± 0.01, *p* = 0.006) and BMI z-scores (0.09 ± 0.03, *p* = 0.001).

### Regression model for prevalent abdominal obesity, overweight and obesity at follow-up

Table 3 shows the results of the generalised linear mixed regression analysis for the possible influences of skipping breakfast, and the overconsumption of soft drinks and screen media on abdominal obesity, overweight and obesity at follow-up. Adjustments were made for socio-economic (migration background, household income, and family education level) and individual (age and gender) variables, assignment to intervention or control group of the underlying program evaluation, and school.Skipping breakfast and the overconsumption of screen media were more highly associated with abdominal obesity (odds ratio 3.36 and 2.46, respectively). Children who skipped breakfast and those who over-consumed screen media at baseline were more likely to be overweight at follow up (odds ratio 2.30 and 2.28, respectively).

**Table 3:** Generalised linear mixed regression model for abdominal obesity, overweight and obesity at follow-up

		Unadjusted		Adjusted*		
	Missing Values	OR	95% CI	OR	95% CI	*p-*value
		Abdominal obesity				
				(n = 1253)		
Skipping breakfast	196	3.36	(2.23; 5.07)	*2.06*	*(1.23; 3.47)*	0.006**
Soft drinks > 1 time per week	198	1.78	(1.22; 2.61)	1.46	(0.92; 2.32)	0.108
Screen media > 1 h/day	1	2.46	(1.76; 3.45)	*2.00*	*(1.23; 3.23)*	0.005**
		Overweight				
				(n = 1251)		
Skipping breakfast	201	2.30	(1.54; 3.45)	*1.71*	*(1.04; 2.80)*	0.034*
Soft drinks > 1 time per week	203	1.65	(1.16; 2.35)	1.29	(0.84; 1.96)	0.246
Screen media > 1 h/day	6	2.28	(1.67; 3.13)	*2.01*	*(1.33; 3.03)*	0.001***
		Obesity				
				(n = 1251)		
Skipping breakfast	201	1.81	(0.94; 3.47)	0.90	[0.39; 2.07)	0.799
Soft drinks > 1 time per week	203	1.80	(1.04; 3.11)	1.57	(0.82; 3.03)	0.177
Screen media > 1 h/day	6	2.16	(1.34; 3.49)	1.87	(0.96; 3.67)	0.068

* adjusted for school, migration background, family education level, household income, age, gender, participation in the intervention, OR Odds Ratio, CI Confidence Interval

*** *p*< 0.001, ** *p*< 0.01, * *p*< 0.05

### Missing data

Children whose records contained missing data were significantly more likely to have a history of migration in their backgrounds, and were significantly more likely to be overweight, obese, or abdominally obese than children whose records contained complete data. On average, children whose records had missing data weighed less than children whose records contained complete data. Children whose records had missing data were more often living in single-parent homes and were more often in homes with a household income less than or equal to €1750. Furthermore, children whose records lacked complete data were less likely to live in a home with a tertiary family education level and were less likely to have been breastfed than were children whose records were complete. Moreover, children whose records had missing data spent more time with a PC on school days and were more likely to skip breakfast than their counterparts.

A sensitivity analysis was added to investigate possible differences between complete case analysis and analysis of datasets comprising multiple imputations. The results are shown in Table 4.

**Table 4:** Differences between analyses with datasets containing complete data (CD) and imputed data (ID)

		Skipping breakfast		Soft drinks > 1 time per week		Screen media > 1h/day	
		OR	95% CI	OR	95% CI	OR	95% CI
Abdominal obesity	CD	*2.06*	(1.23; 3.47)**	1.46	(0.92; 2.32)	*2.00*	(1.23; 3.23)**
	ID	*1.87*	(1.19; 2.96)**	1.37	(0.92; 2.04)	*1.81*	(1.19; 2.75)**
Overweight	CD	*1.71*	(1.04; 2.80)*	1.29	(0.84; 1.96)	*2.01*	(1.33; 3.03)***
	ID	*1.60*	(1.02; 2.50)*	1.38	(0.94; 2.01)	*1.76*	(1.18; 2.61)**
Obesity	CD	0.90	(0.39; 2.07)	1.57	(0.82; 3.03)	1.87	(0.96; 3.67)
	ID	1.02	(0.50; 2.07)	1.56	(0.87; 2.80)	1.65	(0.90; 3.01)
		*B* (SE)	*p*-value	*B* (SE)	*p*-value	*B* (SE)	*p*-value
Changes in WHtR^a,b,c^	CD	*0.50 (0.19)*	0.007**	-0.01 (0.15)	0.966	0.29 (0.16)	0.074
	ID	*0.51 (0.17)*	0.003**	-0.07 (0.13)	0.600	0.17 (0.15)	0.240
Changes in weight [kg]^b,d^	CD	*0.39 (0.12)*	0.001***	-0.08 (0.09)	0.385	0.19 (0.10)	0.054
	ID	*0.51 (0.17)*	0.003**	-0.07 (0.13)	0.600	0.17 (0.15)	0.240
Changes in BMI percentiles^b,e^	CD	*2.01 (0.90)*	0.027*	-0.75 (0.70)	0.282	0.70 (0.78)	0.373
	ID	*2.58 (0.83)*	0.002**	-1.12 (0.64)	0.083	0.21 (0.72)	0.780

OR odds ratio, CI confidence interval, *B* (SE) *B* regression coefficient (standard error), ^a^ multiplied by 10^2^ for better interpretability, badjusted for school, migration background, family education level, household income, age, gender, participation in the intervention, and ^c^baseline WHtR, ^d^baseline weight, ^e^baseline BMI percentiles

*** *p*< 0.001, ** *p*< 0.01, * *p*< 0.05

## Discussion

This study shows that children skipping breakfast experience increased changes in WHtR, weight and BMI measures. Skipping breakfast and the overconsumption of screen media at baseline contributed to abdominal obesity at follow-up. Skipping breakfast and the overconsumption of screen media also influenced overweight at follow-up. No significant associations were found for the consumption of soft drinks with longitudinal weight development or weight status at follow-up.

### Obesity and abdominal obesity

Children participating in the present study were identified to be abdominally obese according to the threshold of WHtR ≥0.5. From these children, 18% were of normal weight, based on the BMI definition. This is in line with recent research literature saying that a considerable number of people are of normal or low weight according to the BMI definition, but are abdominally obese with a higher risk of mortality [30, 31]. For children, rising numbers of abdominal obesity were detected, while rates of overweight and obesity, defined by BMI, seemed to stabilize, thus underestimating changes in weight development [32]. These results correspond with data which show that BMI fails to identify obesity in more than a quarter of children [33]. While BMI measures the general body structure as relative weight for height, WHtR provides information about body fat distribution.

To our knowledge, the majority of studies examine selected parameters, e.g. the association of skipping breakfast, or soft drink consumption or screen media consumption with predominantly one weight parameter, mostly BMI. The present study considers the influence of these critical behaviours on the longitudinal development in WHtR, weight and BMI measures and the presence of abdominal obesity, obesity and overweight at follow-up. Due to the short observation period of one year, and because of the rather gradual development of obesity, results were not as clearly significant as expected, especially for obesity at follow-up. Furthermore, yet not statistically significant, the intervention may have influenced the results. Another reason may be the relatively small number of obese children at follow-up that inhibits proof of significance. Therefore, longer observation periods are necessary to detect further associations.

### Skipping breakfast

Results of the present study are consistent with previous research. Eating behaviours such as consuming unhealthy food or skipping breakfast in children have been reported to be associated with higher odds for overweight [34]. A recent study showed that skipping breakfast was one modifiable factor for developing abdominal obesity in primary schoolchildren [27]. In a study with overweight Latino youth, Alexander et al. reported that higher visceral adiposity was associated with skipping breakfast [35].

### Screen media consumption

Chaput et al. show that sedentary behaviour is associated with higher BMI, weight gain, and obesity in children [36]. Children’s usage time of computers or TVs is increasing, and is associated with adverse health outcomes such as overweight or obesity [37]. Moreover, children having a TV in their bedroom are more likely to have sleep problems and long-term negative consequences on their health [38]. In our study it can be supposed that the identified effects intensify if enlarging the observational period, and children grow older. A study of American schoolchildren found that children who had screen media times of ≥2 h/day had double the odds of being overweight than do children with < 2 h/day [37]. In the present study, we were able to show that already > 1 h/day screen time is sufficient for having at least twice the odds for becoming overweight or abdominally obese.

### Soft drink consumption

In general literature, there is no doubt about the positive association between soft drinks and overweight in children [13–15]. However, no significant association between soft drink consumption and weight development was found in the present study, this may be due to the young age of the children and the generally low consumption of soft drinks in this sample. In preschool children, Newby et al. also found no association between soft drink consumption and changes in weight and BMI [39]. They speculate that the low intakes and limited variations of soft drink consumption limited the results [39]. Low intakes could also be one reason for not finding significant results in the present study, as in primary schools vending machines are not as widespread as in secondary schools and the availability, and thus the consumption of soft drinks, is automatically reduced. Additionally, providing water to children in primary schools is widespread. Besides, Baden-Württemberg is a wealthy federal state with lower rates of social inequality and overweight than other parts of the country. Another reason possibly lies in the way soft drink consumption was assessed, and parents may have replied to the questionnaire in a socially desired manner. The questionnaire did not give information about the frequency of consumption of fruit nectars and of flavoured or chocolate milk drinks that contain high amounts of added sugar. Overall, soft drink consumption was very limited in this sample.

### Implications for families, future interventions and decision makers

Accordingly, interventions influencing positive weight status in schoolchildren have to include lifestyle patterns, such as having regular breakfast, and a responsible consumption of screen media and soft drinks. First of all, parents should be informed about the advantages and importance of a healthy lifestyle, and health-conscious behaviour. Second, institutions such as schools should be involved in the behaviour change. Finally, for obesity prevention, policy makers have to note that healthy eating and lifestyle habits are required at all times, but the cornerstone has to be laid early.

Parents who demonstrate and offer their children healthy and regular breakfast habits fulfil their function as role models. At institutional level, schools that ensure daily breakfast consumption at the start of the school day will reach all children and avoid the problem of skipping breakfast [40]. On a political level, the time of the start of school day should be discussed: A later start of classes might allow families to have breakfast together.

One possible idea for the prevention of overconsumption of screen media is to define determined times of the day for playing computer games or watching TV that regulate the duration of daily media consumption, e.g. in the form of an agreement between parents and children [41]. At all times, health-promoting programmes should offer and enhance various options against using screen media for schoolchildren. Thus, children’s decision making-ability will be strengthened and children will learn and internalize a healthy lifestyle for permanent appropriation [42]. Times of sedentary behaviour are to be replaced with active and meaningful leisure activities.

One promising approach in the reduction of soft drink consumption is being practised via schools. The removal of soft drink vending machines limits the availability of these drinks as well as limiting their consumption by children [43]. One possibility is the installation of water dispensers in schools, or offering water or unsweetened tea for free in classrooms. Furthermore, in primary school, children’s parents should be involved: The regular provision of water, organized by parents, constitutes a suitable measurement for changing the environment.

### Strengths and limitations

This study provides valuable insights into the connection between skipping breakfast, soft drink and screen media consumption with weight development in schoolchildren. There are some strengths and limitations that should be taken into consideration when interpreting these results. One strength of this research is the strict protocol of a longitudinal trial. A second strength is the large sample size and the fact that the study includes data from an entire state of Germany, although the study is not representative for the whole of Germany. All anthropometric data were objectively measured by trained staff in a standardized procedure and are of high quality. Furthermore, the Institute of Epidemiology and Medical Biometry at Ulm University managed data professionally, and advised in statistical issues. Finally, to our knowledge, this the first study to specifically investigate these three weight-influencing parameters in primary schoolchildren in combination. However, there are also some limitations: First, the observational character of the study may have led to some biased results. Due to the young age of the children, parental report measures were used to assess health and lifestyle characteristics, and some of the questions might have been answered in a socially desired way or show the Hawthorne effect, which describes that participants in observational studies behave differently. Moreover, the investigated variables on children’s weight development could have been complemented by chronobiological aspects. Information on the children’s sleep was not collected, but may also be relevant for their health [44]. As far as school schedules as a further influencing factor on chronobiological aspects are concerned, the included primary schools started between half past seven and eight o’clock and included one or two break times per morning. At the time of the assessment in 2010, all-day school was not yet very common in primary schools in Baden-Württemberg, so most children went home after school at noon.

Furthermore, participation in this study was voluntary and only teachers and parents who gave their agreement were included. Thus, it seems reasonable that teachers and parents who were motivated and health conscious were more likely to take part. Parental breakfast and soft drink intake were not assessed in the present investigation, but in future research these parameters should be included. Another problem of observational studies are missing values which may, in the worst case lead to biased results [26]. Therefore, a missing data analysis and additionally a sensitivity analysis with imputed data were performed. The latter confirmed the significance of the investigated influence of skipping breakfast and screen media use on weight development.

## Conclusion

Soft drink consumption was not associated with weight status in this sample, but should be investigated in more detail in future research. The skipping of breakfast and the overconsumption of screen media influence weight development in primary schoolchildren. Dietary improvements and restriction in screen times are promising approaches in obesity prevention in schoolchildren. Especially with regard to the high prevalence of overweight and abdominal obesity in parents, healthy breakfast habits both at home or in schools and an awareness of screen media consumption may not only improve children’s health but that of their parents, too. Children, parents, schools and governments should be involved in behavioural and structural prevention. Finally, further research should examine the combined effects of these crucial variables on weight development for a longer period, at least over the period of four school years in primary school.

## Abbreviations

BMI: Body mass index
CASMIN classification: Comparative Analysis of Social Mobility in Industrial Nations
DRKS: German Clinical Trials Register
ISAK: International Standards for Anthropometric Assessment
KiGGS survey: German Health Interview and Examination Survey for Children and Adolescents
NCD: Non-communicable diseases
OR: Odds ratio
WC: Waist circumference
WHO: World Health Organization
WHtR: Waist-to-height ratio
